# Development of a rapid cell-fusion-based phenotypic HIV-1 tropism assay

**DOI:** 10.7448/IAS.16.1.18723

**Published:** 2013-09-18

**Authors:** Phairote Teeranaipong, Noriaki Hosoya, Ai Kawana-Tachikawa, Takeshi Fujii, Tomohiko Koibuchi, Hitomi Nakamura, Michiko Koga, Naoyuki Kondo, George F Gao, Hiroo Hoshino, Zene Matsuda, Aikichi Iwamoto

**Affiliations:** 1 Division of Infectious Diseases, Advanced Clinical Research Center, Institute of Medical Science, University of Tokyo, Tokyo, Japan; 2 Department of Infectious Disease Control, International Research Center for Infectious Diseases, Institute of Medical Science, University of Tokyo, Tokyo, Japan; 3 Department of Infectious Diseases and Applied Immunology, Affiliated Hospital to Institute of Medical Science, University of Tokyo, Tokyo, Japan; 4 Research Center for Asian Infectious Diseases, Institute of Medical Science, University of Tokyo, Tokyo, Japan; 5 Japan–China Joint Laboratory of Structural Virology and Immunology, Institute of Biophysics, Chinese Academy of Sciences, Beijing, China; 6 CAS Key Laboratory of Pathogenic Microbiology and Immunology, Institute of Microbiology, Chinese Academy of Sciences, Beijing, China; 7 Gunma University Graduate School of Medicine, Gunma University, Gunma, Japan

**Keywords:** HIV-1, co-receptor, tropism, co-receptor usage, fusion, chemokine receptor

## Abstract

**Introduction:**

A dual split reporter protein system (DSP), recombining *Renilla* luciferase (RL) and green fluorescent protein (GFP) split into two different constructs (DSP_1–7_ and DSP_8–11_), was adapted to create a novel rapid phenotypic tropism assay (PTA) for HIV-1 infection (DSP-Pheno).

**Methods:**

DSP_1–7_ was stably expressed in the glioma-derived NP-2 cell lines, which expressed CD4/CXCR4 (N4X4) or CD4/CCR5 (N4R5), respectively. An expression vector with DSP_8–11_ (pRE11) was constructed. The HIV-1 envelope genes were subcloned in pRE11 (pRE11-env) and transfected into 293FT cells. Transfected 293FT cells were incubated with the indicator cell lines independently. In developing the assay, we selected the DSP_1–7_-positive clones that showed the highest GFP activity after complementation with DSP_8–11_. These cell lines, designated N4R5-DSP_1–7_, N4X4-DSP_1–7_ were used for subsequent assays.

**Results:**

The env gene from the reference strains (BaL for R5 virus, NL4-3 for X4 virus, SF2 for dual tropic virus) subcloned in pRE11 and tested, was concordant with the expected co-receptor usage. Assay results were available in two ways (RL or GFP). The assay sensitivity by RL activity was comparable with those of the published phenotypic assays using pseudovirus. The shortest turnaround time was 5 days after obtaining the patient's plasma. All clinical samples gave positive RL signals on R5 indicator cells in the fusion assay. Median RLU value of the low CD4 group was significantly higher on X4 indicator cells and suggested the presence of more dual or X4 tropic viruses in this group of patients. Comparison of representative samples with Geno2Pheno [co-receptor] assay was concordant.

**Conclusions:**

A new cell-fusion-based, high-throughput PTA for HIV-1, which would be suitable for in-house studies, was developed. Equipped with two-way reporter system, RL and GFP, DSP-Pheno is a sensitive test with short turnaround time. Although maintenance of cell lines and laboratory equipment is necessary, it provides a safe assay system without infectious viruses. With further validation against other conventional analyses, DSP-Pheno may prove to be a useful laboratory tool. The assay may be useful especially for the research on non-B subtype HIV-1 whose co-receptor usage has not been studied much.

## Introduction

A new class of drugs to combat HIV-1 infection emerged in 2007 with the marketing approval of maraviroc, a small molecule that binds specifically to the CCR5 co-receptor to block viral attachment and entry [1]. While entry inhibitors are a welcome addition to the antiretroviral arsenal, one problem with this new class of drugs is that treatment is effective only against viruses with the specified co-receptor usage. HIV-1 tropism is defined by the chemokine co-receptors used for viral attachment: R5-tropic viruses use CD4/CCR5, X4-tropic viruses use CD4/CXCR4 and R5X4- or dual-tropic viruses use both CD4/CCR5 and CD4/CXCR4 [2–4]. In clinical treatment with maraviroc, the presence of X4- or dual-tropic viruses is associated with treatment failure [5,6], and a tropism assay is mandatory before treatment initiation.

HIV-1 tropism may be examined genotypically or phenotypically. Genotypic tropism assay (GTA) is based on DNA amplification and sequencing of the third variable (V3) region of the envelope glycoprotein gp120, shown by genetic mapping to be the major determinant of HIV-1 tropism [7–10]. GTA has advantages of platform portability, low cost and rapid turnaround time [11]; however, the interpretation of the sequences is complicated because of the high variability [12]. The assay used in association with maraviroc treatment is the phenotypic tropism assay (PTA) Trofile™ (Monogram Biosciences Inc., CA, USA), a CD4 cell culture assay using replication-defective pseudoviruses [13]. Although Trofile™ is considered the gold standard, a simpler and effective PTA would be useful.

Here, we describe a novel, cell-fusion-based PTA that uses a dual split reporter protein system (DSP) [14,15] to measure HIV-1 tropism by both Renilla luciferase (RL) activity and green fluorescent protein (GFP) activity. We validated the DSP-Pheno assay using HIV-1 reference strains and applied the assay to test clinical samples from patients with HIV-1 infection.

## Methods

### Approval of the study and recombinant DNA experiments

Plasma samples from HIV-1-positive patients attending the hospital affiliated with the Institute of Medical Science, the University of Tokyo (IMSUT) were collected and kept frozen until use. Patients provided written informed consent, and the study was approved by the Institutional Review Board of the University of Tokyo (approval number 20-31). Recombinant DNA experiments used in this work were approved by the Institutional Review Board (approval number 08-30), and by the review board in the Ministry of Education, Culture, Sports, Science and Technology (MEXT; approval number 23-1927).

### Cell lines

Cell lines N4, N4X4 and N4R5 are derived from the human glioma NP-2 cell line and stably express CD4, CXCR4 and CCR5, respectively [16,17]. NP-2-derived cell lines were grown in M10+ medium (modified Eagle's medium (MEM; Sigma, St. Louis, MO, USA) supplemented with 10% heat-inactivated foetal bovine serum (FBS), 100 units/ml of penicillin and 0.1 mg/ml of streptomycin). 293FT cells (Invitrogen, Carlsbad, CA, USA) were grown in D10+ medium (Dulbecco's modified Eagle's medium (DMEM, Sigma) supplemented with 10% FBS, 100 units/ml of penicillin and 0.1 mg/ml of streptomycin). All cell cultures were maintained at 37°C in a humidified 5% CO_2_ incubator.

### Reference viral envelopes

The plasmids encoding reference HIV-1 envelopes with well-characterized co-receptor usage were obtained from NIH AIDS Research & Reference Reagent Program (NIH ARRRP: Germantown, MD, USA). NL4-3 and LAI represented X-4 tropic viruses; BaL represented R5-tropic viruses; and SF2 represented dual (R5X4)-tropic viruses. The co-receptor usage of these laboratory strains has been published [13,18–22].

### Construction of DSP expression plasmids

The DSP system utilizes a pair of chimeric reporter proteins, DSP_1–7_ and DSP_8–11_, each of which is a fusion of split green fluorescent protein (spGFP) and split Renilla luciferase (spRL) [15]. DSP_1–7_ fuses the N-terminal region of RL (amino acids 1–229) to the N-terminal region of GFP (amino acids 1–157), with a linker sequence separating the two regions. DSP_8–11_ has the complementary structure, with the C-terminal region of GFP (amino acids 158–231), fused to the C-terminal region of RL (amino acids 230–311), also separated by a linker sequence. When both reporter proteins are present in the same cell, they each recover full activity.

To generate pLenti-DSP_1–7_ plasmid, we first amplified an attB-flanked DSP_1–7_ fragment (1251 bp) using pDSP _1–7_ as a template and attB-flanked primers [attB1-DSP1-1F (56-mer, 5′-GGGGACAAGTTTGTACAAAAAAGCAGGCTGGGCTAGCCACCATGGCTTCCAAGGTG -3′) and attB2-DSP1-1R (51-mer, 5′-GGGGACCACTTTGTACAAGAAAGCTGGGTGCTCTAGATCACTTGTCGGCGG-3′)]. Sequential amplicons were transferred to pDONR-221 and pLenti6.3/V5-DEST (Invitrogen) using the ViraPower™ HiPerform™ Lentiviral Gateway^®^ Expression System (Invitrogen) according to the manufacturer's protocol. Constructs were verified by sequencing.

**Figure 1:**
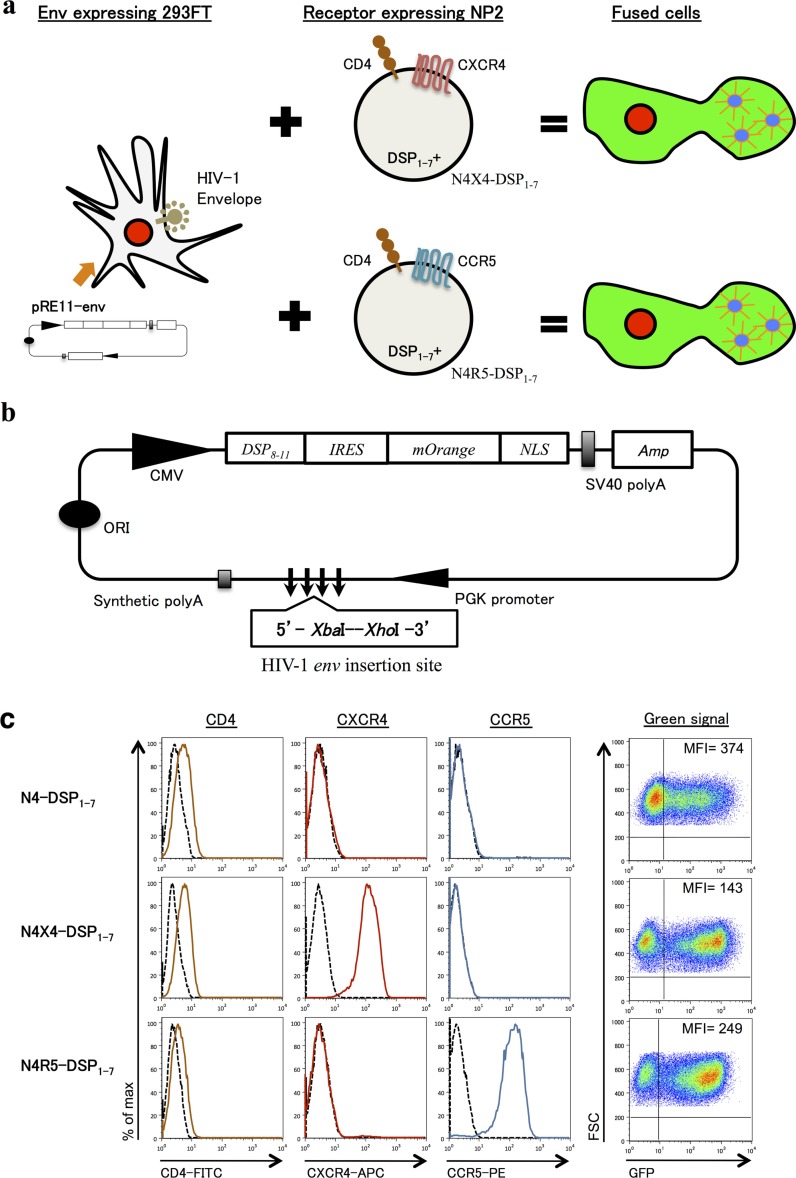
A cell-fusion-based phenotypic tropism assay for HIV-1: DSP-Pheno. (a) Schematic representation of DSP-Pheno assay system. (b) Schematic representation of pRE11, an expression vector for HIV-1 env and DSP_8–11_. pRE11 encodes also mOrange with a nuclear localization signal as an indicator of transfection. (c) NP-2-derived clones stably expressing DSP_1–7_ (N4-DSP_1–7_, N4X4-DSP_1–7_ and N4R5-DSP_1–7_) were selected by the high GFP expression after direct transfection of pDSP_8–11_. The expression of CD4/co-receptors was reconfirmed by appropriate monoclonal antibodies and FACS analysis.

An expression vector, pRE11 (Figure 1), was constructed for the co-expression of DSP_8–11_ and HIV-1 *env* by multiple rounds of PCR and subcloning. Source plasmids were pIRES2-AcGFP1 (Clontech), pmOrange (Clontech), pDSP_8–11_ [15] and pmirGLO (Promega). pRE11 incorporated multiple cloning sites under the PGK promoter for the insertion of HIV-1 *env* (Shown as 5′-XbaI-XhoI-3′ in Figure 1b). Necessary restriction enzyme cleavage sites used for construction, including multiple cloning sites (XbaI-MluI-SwaI-AgeI-XhoI), were created using synthetic oligonucleotides and PCR. A CMV promoter drives pDSP_8–11_ directly. The same CMV promoter expresses mOrange with a nuclear localization signal that serves as a marker for successful transfection via internal ribosomal entry site (IRES). All PCR fragments were confirmed by sequencing.

### NP-2-derived fusion indicator cell lines

We used the ViraPower Packaging Mix with Lipofectamine 2000 (Invitrogen) to transfect 293FT cells with pLenti-DSP_1–7_ and create pseudoviruses containing the DSP_1–7_ expression cassette (Lenti-DSP_1–7_). We next infected cell lines NP-2/CD4 (N4), CD4/CXCR4 (N4X4) and CD4/CCR5 (N4R5) with pseudoviruses containing LentiDSP_1–7_ for 2 hours. Cells were distributed in 96-well tissue culture plates at a density of 75 cells/plate (0.8 cell/well) and grown in the presence of 4 µg/ml blasticidin. Approximately 50 candidate clones from each cell line were randomly selected and tested for FITC intensity using FACS Calibur (BD Biosciences, Franklin Lakes, NJ, USA) 48 hours after transfection of pDSP_8–11_. FACS data were analyzed by Flow Jo version 8.7.1 (Tree Star Inc., OR, USA). Clones with the highest median FITC intensity were expanded in M10+ supplemented with 4 µg/ml of blasticidin (M10 + 4) for further assays.

### Generation of pRE11-env strains

Full-length HIV-1 *env* was prepared by PCR amplification from clinical plasma samples as described [23]. Viral RNA was extracted from 140 µl of patient's plasma by QIAamp Viral RNA Mini kit according to the manufacturer's recommendation (QIAGEN, Hilden, Germany). One-step RT-PCR using SuperScript III and High Fidelity Platinum^®^ Taq DNA polymerase (Invitrogen) was carried out in five separate 15-µl reactions to minimize the bias created by PCR. The reaction mixture contained 2 µl of RNA template, 7.5 µl of 2× reaction buffer, 0.3 µl of 5 mM MgSO_4_, 0.3 µl of each 10 µM of forward primer (Env-1F, 25-mer, 5′-TAGAGCCCTG GAAGCATCCAGGAAG-3′) and reverse primer (Env-3Rmix, equimolar mixture of 30-mer, 5′ -TGCTGTATTGCTACTTGTGATTGCTCCATA-3′ and 30-mer, 5′ -TGCTGTATTGCTA CTTGTGATTGCTCCATG-3′), 0.6 µl of SuperScript III and High Fidelity Platinum^®^ Taq DNA polymerase, 0.25 µl of RNAse OUT and 3.75 µl of nuclease-free water with the final volume of 15 µl/reaction. The one-step RT-PCR condition was 55°C for 30 minutes, 94°C for 2 minutes followed by 30 cycles of 94°C for 20 seconds, 55°C for 30 seconds, 68°C for 4 minutes, then extension at 68°C for 5 minutes. The fragment by the first-round amplification extended from NL4-3 reference position of 5853–8936. Products from five independent reactions were combined. Four microliters of the mixed first-round PCR products were used as the template for each of five independent second-round PCR reactions employing EnvB-2F-Xba (41-mer, 5′-TAGCTCTAGAACGCGTCTTAGGCATCTCCTATGGCAG GAAG-3′) and EnvB-4R-Xho (41-mer, 5′ -TAGCCTCGAGACCGGTTACTTT TTGACCACTTGCCACCCAT-3′) as the forward and reverse primers, respectively. The second PCR was carried out according to the standard 50-µl PCR protocol of the Platinum^®^ PCR SuperMix High Fidelity as described above. The fragments amplified by the second PCR extended from NL4-3 reference position of 5957–8817. After digestion with Xba I and Xho I, about 3-kb PCR products were purified by 1.2% agarose gel and QIAquick gel extraction kit (Qiagen). The purified products were inserted into pRE11 at XbaI and XhoI sites, resulting in HIV-1*env* expression plasmid (pRE11-envbulk) from each patient. pRE11-envbulk, representing a quasispecies of *env* population from each patient, was prepared by transfecting into *E. coli* JM109. For bulk analysis, transfected JM109 was expanded to 25 ml, followed by QIAGEN Plasmid Midi Kit (Qiagen) for DNA extraction.

### Cell-fusion assay

On the day before transfection, 500 µl aliquots of 293FT cells in DMEM supplemented with 10% FBS (D10) were seeded in 24-well tissue culture plates at a density of 2.8×10^5^ cells/well and incubated overnight to 70–80% confluency. The cells were then transfected with pRE11-envstrain or pRE11-envbulk according to the manufacturer's protocol (Roche). On the same day, 100 µl aliquots of N4-DSP_1–7_, N4X4-DSP_1–7_ and N4R5-DSP_1–7_ cells in MEM supplemented with 10% FBS (M10) were seeded in a 96-well tissue culture, optical bottom plate (NUNC, Thermo Fisher Scientific Inc., NY, USA) at a density of 1×10^4^ cells/well and incubated at 37°C. Forty-eight hours after transfection, the medium of transfected 293FT cells was removed by aspiration and replaced with 1 ml of PBS (Sigma) at RT. Transfected 293FT cells were resuspended by gentle pipetting.

To start the cell-fusion assay, 150 µl/well of transfected cells were overlaid onto N4-DSP_1–7_, N4X4-DSP_1–7_ and N4R5-DSP_1–7_ cells. The cells were incubated for fusion at 37°C in a humidified 5% CO_2_ incubator for 6 hours, and then analyzed by automatic image capture using an In Cell Analyzer 1000 (GE Healthcare). Four fields/well of image were captured through red, green and bright field channels, and fused cells were identified by the presence of two or more red nuclei surrounded by a green area (cytoplasm). Immediately after image capturing, EnduRen™ Live Cell Substrate (Promega) was added to each well, and luciferase activity was measured three times using a Glomax 96 microplate luminometer (Promega), according to the manufacturer's instructions. The mean luciferase activity, recorded as relative light unit (RLU), was the average of three measurements per well. The experiments were conducted in triplicates and repeated independently at least three times.

To test the co-receptor specificity, 2 µM/well of the appropriate inhibitor was added to the cells 90 minutes prior to the cell-fusion assay (CXCR4 inhibitor AMD3100 (Sigma) to N4X4-DSP_1–7_ cells and CCR5 inhibitor maraviroc (Sigma) to N4R5-DSP_1–7_ cells).

### Genotyping

pRE11-envbulk were sequenced in both the 5′ and 3′ directions using population-based sequencing on the ABI 3130xl genetic analyzer (Applied Biosystems, Foster City, CA, USA) using BigDye Terminator V3.1 (Applied Biosystems) with forward primer E110 (5′ -CTGTTAAATGGCAGTCTAGCAGAA-3′), and reverse primer Er115 (5′ -AGAAAAATTCCCCTCCACAATTAA-3′). The V3 nucleotide sequences were submitted to the Geno2Pheno (co-receptor) algorithm (http://coreceptor.bioinf.mpi-inf.mpg.de) setting the false positive rate (FPR) at 10%.

## Results

### Construction of DSP_1–7_ and DSP_8–11_ expression vectors

We inserted DSP_1–7_ or DSP_8–11_ sequences into blasticidin-resistant lentivirus vectors and then infected NP-2/CD4 (N4), NP-2/CD4/CXCR4 (N4X4) and NP-2/CD4/CCR5 (N4R5) cells with the recovered pseudoviruses, selecting for blasticidin-resistant clones. We screened 49, 51 and 43 lentivirus-infected and blasticidin-resistant clones from N4, N4X4 and N4X5, respectively, for high levels of DSP_1–7_ or DSP _8–11_ expression following super-transfection with the complementary plasmid (pDSP_8–11_ or pDSP_1–7_, respectively). From each of the cell lines, we selected the blasticidin-resistant and DSP_1–7_-positive clone that showed the highest GFP activity after complementation with DSP_8–11_. These cell lines, designated N4-DSP_1–7_, N4X4-DSP_1–7_ and N4R5-DSP_1–7_, were re-evaluated for their expression of CD4, CXCR4 and CCR5 on the cell surface (Figure 1c).

Using this approach, we obtained N4- and N4R5-cells expressing high levels of DSP_8–11_, but were unable to obtain a stable N4X4 cell line expressing DSP_8–11_ (data not shown). To circumvent this problem, we decided to generate 293FT cells transiently expressing both DSP_8–11_ and the HIV-1 *env* protein and develop a cell-fusion assay system using those cells together with the NP-2-derived cells stably expressing DSP_1–7_ (Figure 1a). Thus, we constructed the expression vector pRE11, containing the DSP_8–11_ expression cassette and cloning sites for insertion of HIV-1 *env* sequences under the control of the PGK promoter (Figure 1b).

### Validation of the cell-fusion assay using the *env* gene from laboratory HIV-1 strains

**Figure 2:**
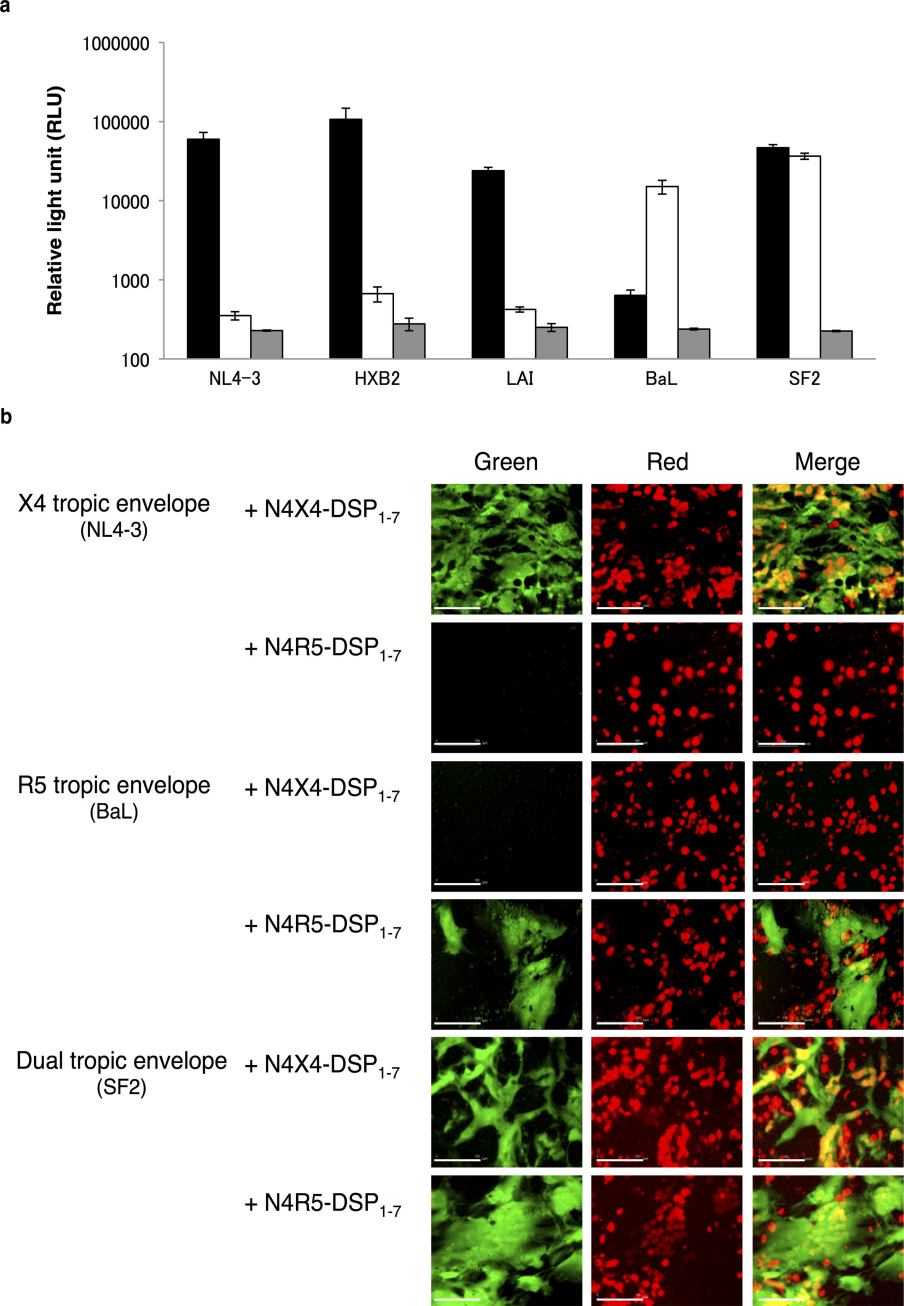
Validation of the cell-fusion assay using env genes from HIV-1 reference strains. (a) RL activities after cell fusion were measured using NL4-3, HXB2 and LAI as X4 reference strains while BaL or SF2 as R5 or R5X4 strains, respectively. Black columns: RL activities on N4X4-DSP_1–7_. White columns: activities on N4R5-DSP_1–7_. Grey columns: activities on N4-DSP_1–7_. Small bars at the top of each column indicate the mean RLU±SD from three independent experiments. (b) Successful cell fusion is indicated by the green fluorescence in the cytoplasm. Red spots were mOrange activity in the nuclei showing successful transfection. Merged images showed multinuclear cells with multiple yellow/orange nuclei surrounded by green cytoplasm.

We validated the DSP assay system (DSP-pheno) using pRE11 constructs engineered to contain *env* sequences from reference strains with known co-receptor usage. The *env* reference constructs, which also contained the DSP_8–11_ expression cassette, were the following: pRE11-HXB2, pRE11-LAI, and pRE11-NL4-3 (X4 strains); pRE11-BaL (R5 strain); and pRE11-SF2 (dual strain). The cell-fusion assays were performed with N4X4-DSP_1–7_ or N4R5-DSP_1–7_ cells in combination with 293FT cells transiently expressing one of the pRE11-env constructs. In all the assays, both RL and GFP activities were restored only when cells expressing the appropriate *env* and co-receptor combinations were co-cultured (Figure 2a and b). Co-culture of N4X4-DSP_1–7_ or N4R5-DSP_1–7_ in combination with the 293FT cells transiently expressing the pRE11-env constructs of discordant tropism served as a negative control for expression of RL activities (Figure 2a) and GFP signals (Figure 2b). The absence of RL activities on N4-DSP_1-7_ confirmed the importance of co-receptors for the fusion.

### Assay detection thresholds and sensitivity for minor populations

**Figure 3:**
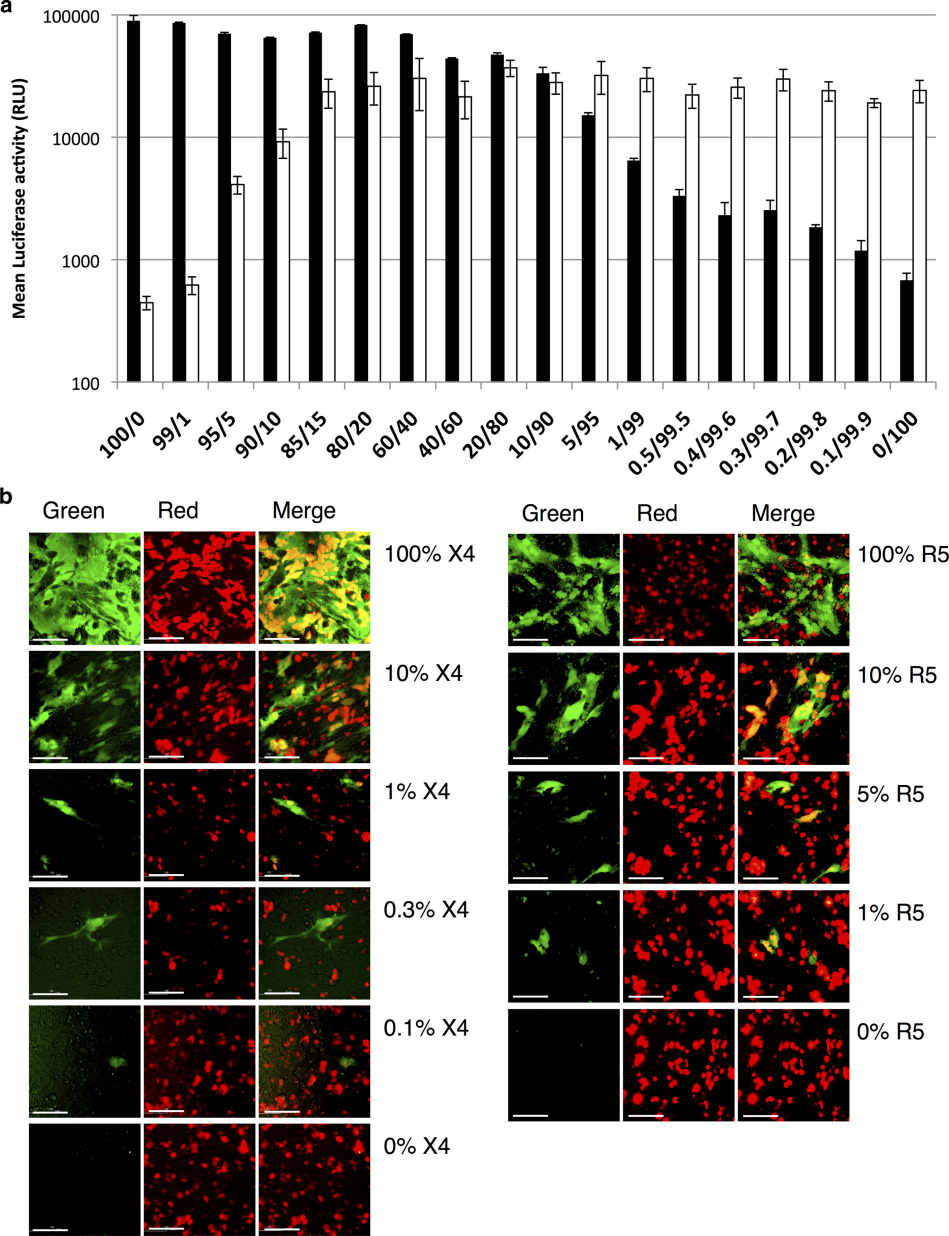
Assay sensitivities for minor populations. pRE11-NL4-3 (X4) and pRE11-BaL (R5) were mixed at indicated ratios. The total plasmid concentration in the mixture was adjusted to 100 ng/µl. The mixture was transfected to 293FT cells and fused to the indicator cells. (a) RL activities. Black columns show the RL activities on X4 indicator (N4X4-DSP_1–7_), while white columns on R5-indicator (N4R5-DSP_1–7_). Small bars at the top of each column indicate the mean RLU±SD from three independent experiments. (b) GFP activities. Left panel shows activities of the mixture with indicated ratio on X4 indicator (N4X4-DSP_1–7_), while right panel on R5-indicator (N4R5-DSP_1–7_).

To evaluate assay sensitivity in identifying minor variants within a single sample, we mixed pRE11-NL4-3 (X4) and pRE-BaL (R5) in varying ratios and measured RL activities and GFP signals (Figure 3a and b). Both methods of detection identified X4 viruses more readily than R5 viruses. Based on luciferase activity, the presence of approximately 0.3% X4 viruses gave values significantly higher than background (0% X4), while R5 viruses had to comprise approximately 5% of the mixture for the signal to be detectable over background (Figure 3a). Similarly, based on GFP signals, X4 viruses comprising as little as 0.1% of the mixture could be detected, while detection of R5 viruses had a minimum threshold of approximately 1% (Figure 3b).

### Validation of the chemokine receptor specificity using the CXCR4 inhibitor AMD3100 and CCR5 inhibitor maraviroc

**Figure 4:**
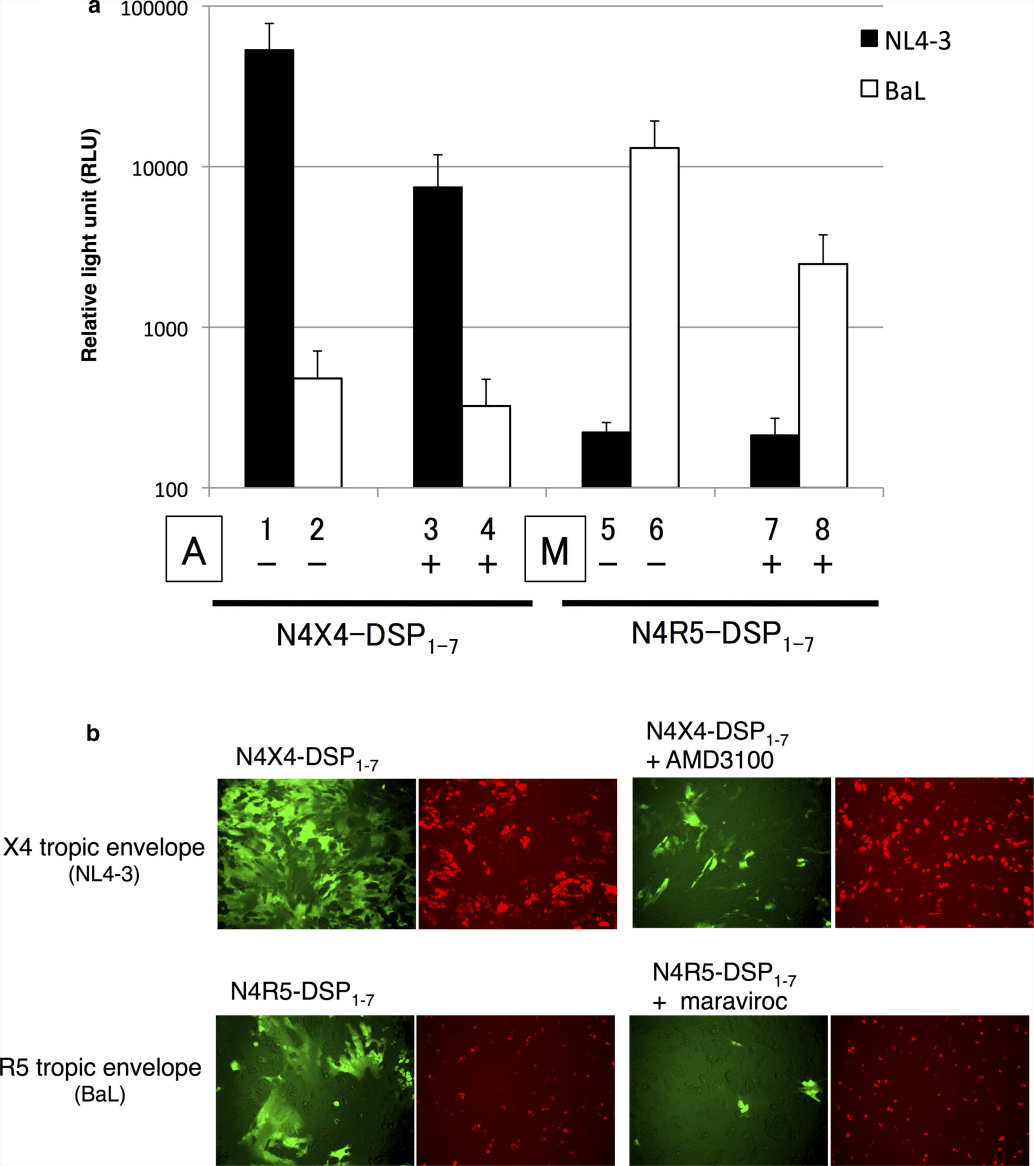
Inhibition of cell fusions by entry inhibitors. Two µM/well of CXCR4 inhibitor AMD3100 or CCR5 inhibitor maraviroc were added into N4X4-DSP_1–7_ and N4R5-DSP_1–7_ cells 90 minutes prior to cell-fusion assay using env derived from reference strains.(a) RL activities. Columns show the mean RLU±SD from 5 independent experiments. Black columns, RL activities of env derived from X4 reference strain (NL4-3); white columns, RL activities of env derived from R5 reference strain (BaL). Results from X4-indicator (N4X4-DSP_1–7_) (lanes 1–4) and R5-indicator (N4R5-DSP_1–7_) (lanes 5–8). A, AMD3100; M, maraviroc. Presence or absence of inhibitor indicated by + or −, respectively. (b) GFP activities. Green fluorescence in the left panel of each pair shows successful cell fusions; red spots in the right panels show the successful transfection. Reference strains, indicator cells and inhibitors used are shown in the figure.

293FT cells expressing *env* from reference strains NL4-3 (X4) or BaL (R5) were co-cultured with N4X4-DSP_1–7_ or N4R5-DSP_1–7_ cells in the absence or presence of AMD3100 or maraviroc (Figure 4a and b). In the absence of inhibitors, RL activities of the matched co-culture were high (Figure 4a). In the presence of AMD3100, the RL activity of the co-culture of 293FT cells expressing NL4-3-derived *env* with N4X4-DSP_1–7_ cells was reduced by 83%. The RL activity of the co-culture of 293FT cells expressing BaL-derived *env* with N4X4-DSP_1–7_ cells was low in the absence of AMD3100 and was not affected significantly by its presence. The RL activity of the co-culture of 293FT cells expressing BaL-derived *env* with N4R5- DSP_1–7_ was reduced by 81% in the presence of maraviroc. The RL activity of the co-culture of 293FT cells expressing NL4-3-derived *env* with N4R5- DSP_1–7_ was low regardless of the presence or absence of maraviroc. The results indicated that DSP-Pheno could be used as an assay for entry inhibitors.

### Cell-fusion assay of clinical samples

**Figure 5:**
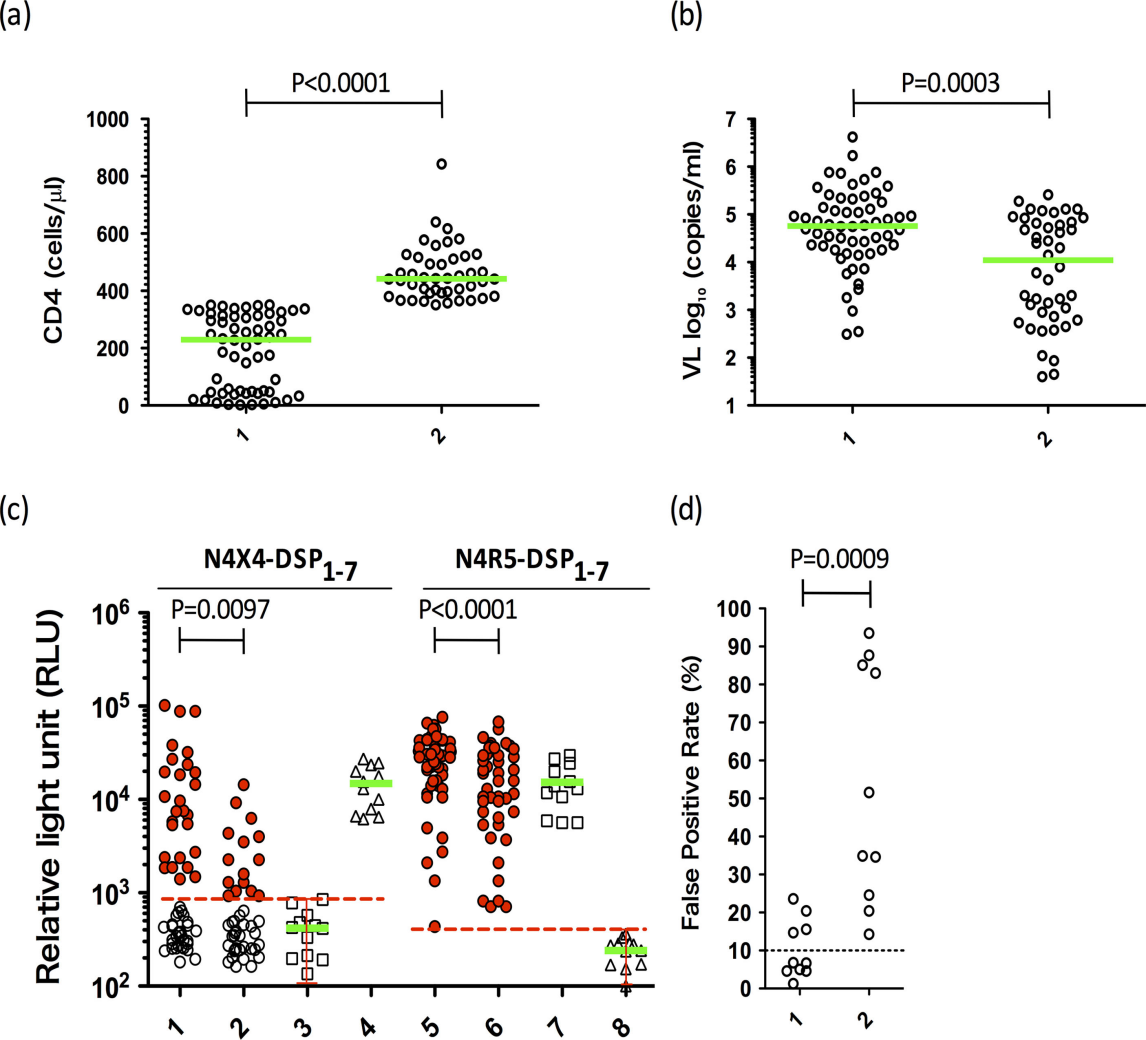
DSP-Pheno and Geno2Pheno on clinical samples. Patients were assigned to one of the two groups based on CD4+ T cell counts. Horizontal green bars indicate the median value. (a) CD4 counts of the patients. Lane 1, Fifty-seven patients with CD4<350 cells/µl, median=228 (range 2–350) cells/µl. Lane 2, Forty-four patients with CD4>350 cells/µl, median 442 (range 351–843) cells/µl. (b) Viral load of each group. Lane 1, CD4<350 group, median viral load=4.77 (range 2.97–6.62) log10 copies/ml. Lane 2, CD4<350 group, median viral load=4.04 (range 1.60–5.41) log 10 copies/ml. (c) Mean luciferase activities of the patients’ plasma samples. Lanes 1 and 5, CD4<350 group; lanes 2 and 6, CD4>350 group; lanes 3 and 7, R5 controls (BaL); lanes 4 and 8, X4 controls (NL4-3). Dashed red lines are the cut-off value, that is, the mean value+2SD based on 3 three determinations in 12 independent experiments for each combination of negative control and indicator cell. (d) Geno2Pheno [co-receptor] analysis of representative samples. Lane 1, 10 samples from dual/X4 [N4X4-DSP_1–7_ (+), N4R5-DSP_1–7_ (+)] group by DSP-Pheno; Lane 2, 10 samples from R5 [N4X4-DSP_1–7_ (−), N4R5-DSP_1–7_ (+)] group by DSP-Pheno. For dual/X4 and R5 group, five patients each from CD4 < 350 and CD4 >350 groups were chosen. Dashed line indicates the cut-off value as 10% of FPR.

To evaluate assay performance using clinical samples, we selected plasma samples from 101 treatment-naïve, HIV-1-positive patients, whose infection with clade B viruses had been confirmed (data not shown). The patient population was classified into two groups based on CD4 T cell count. The low CD4 group consisted of 57 patients with CD4 T cell counts <350 cells/µl; median 228 (range 2–350) cells/µl, and median viral load was 4.77 (range 2.97–6.62) log 10 copies/ml (Figure 5a and b). The high CD4 group consisted of 44 patients with CD4 cell counts >350 cells/µl; median 442 (range 351–843) cells/µl, and median viral load was 4.04 (range 1.60–5.41) log 10 copies/ml. The viral load differences between the two groups were statistically significant by the Mann–Whitney *U* test (*p*<0.001). Aliquots of viral envelope DNA from each plasma sample were used to construct pRE11-envbulk for transfection into 293FT cells. The plasma viral load necessary for the assay was roughly 3.00 log 10 copies/ml for subtype B viruses, although we could amplify the env gene in a patient with 1.60 log 10 copies/ml.

We used the laboratory strain, BaL as the R5 control and NL4-3 as the X4 control to define the cut-off values. We examined BaL on N4X4-DSP_1–7_ cells and NL4-3 on N4R5-DSP_1–7_ cells. We defined the cut-off value tentatively as the mean value+2SD based on 3 determinations in 12 independent experiments for each combination of negative control and indicator cell (red dashed line in Figure 5c). As expected, both combinations showed stably low RL activities, with cut-off values of 876 for N4X4-DSP_1–7_ cells and 397 RLU for N4R5-DSP_1–7_ cells.

Samples from all patients gave positive RL signals on R5 indicator cells (N4R5-DSP_1–7_) in the fusion assay, which suggested that the bulk of virus in each patient was able to use CCR5 as the co-receptor (Figure 5c, lanes 5 and 6). Median RLU value of the low CD4 group was significantly higher than that of the high CD4 group on R5 indicator cells (*p*<0.0001). Median RLU value of the low CD4 group was also higher significantly on X4 indicator cells (*p*=0.0097) and 26/57 (46%) of low CD4 cases versus 15/44 (34%) of high CD4 cases gave positive RL signals (Figure 5c, lanes 1 and 2). Higher fusion activities on both indicator cells are compatible with higher viral loads in patients with lower CD4 T cell counts and may suggest more dual or X4 tropic (dual/X4) viruses in this group of patients.

To compare the result with conventional GTA, we selected 10 samples each from dual/X4 [N4X4-DSP_1–7_ (+), N4R5-DSP_1–7_ (+)] and R5 [N4X4-DSP_1–7_ (−), N4R5-DSP_1–7_ (+)] cases. Env V3 nucleotide sequences from pRE11-envbulk plasmids were subjected to the Geno2Pheno [co-receptor]. R5-representative samples showed significantly higher FPR than dual/X4-representative samples (*p*=0.0009) (Figure 5d). DSP-Pheno and Geno2Pheno gave concordant results in 10/10 R5 and 6/10 dual/X4 samples (Figure 5d). Although there were four samples with discordant result in dual/X4 samples, FPR of these samples were low (range: 14.7–23.6%).

## Discussion

We developed a quick, safe and sensitive HIV-1 PTA utilizing double split proteins (DSP-Pheno) and validated the specificity of the assay using laboratory strains with known co-receptor usage. We recognize several limitations of this preliminary study, but the results nevertheless are promising. We assayed bulk envelope genes amplified from plasma from HIV-1-infected patients, rather than cloned envelope genes, and our sample only included subtype B HIV-1. Future studies are necessary to demonstrate the usefulness of the DSP-Pheno.

One caveat of the DSP-Pheno assay is that it is a cell-fusion system, and cell–cell fusion may differ in significant details from virus–cell fusion. For example, recent studies have shown that HIV-1 virions carry fewer surface glycoproteins than previously assumed [24]. The DSP-Pheno assay uses neuroglyoma cell-derived NP-2 cell lines with overexpressed CD4 and co-receptors. Although these NP-2-derived cell lines have been characterized extensively [16,17], some unknown cell surface molecules may be involved in the fusion process. The DSP-Pheno assay is a gag-free system and requires only the assembly of reporter proteins pre-formed in the fusion partner, but infection by a retrovirus requires that the entire gag particle pass through the fusion pore. Careful comparison between DSP-Pheno and in-house pseudoviral assay or GTA using clonal clinical isolates is under way.

GFP portion is necessary as a module of DSP to compensate weak self-association of split RL [15]. Although RL would be more suitable for quantitative assay, GFP may prove single clear positive fusion in the sample with very low RL readout. This feature of DSP-Pheno incorporating two different assays may be useful for certain scientific purposes.

Although several issues remain to be clarified, DSP-Pheno has multiple advantages over the conventional pseudoviral PTA: (i) the turnaround time for DSP-Pheno is short, with results available in as few as 5 days, starting from patients’ plasma; (ii) DSP-Pheno is a virus-free assay that does not require a special biosafety facility, making it particularly appealing for in-house use; and (iii) the RL assay in DSP-Pheno has high sensitivity and specificity and compares favourably with the best pseudoviral PTA published in the detection of minor X4 populations using laboratory strains. Trofile™ (Monogram Biosciences Inc., CA, USA) is currently the only commercially available PTA approved for clinical use, and the latest version, “Enhanced Trofile™,” detects X4 minor populations present in concentrations as low as 0.3% [25]. A pseudoviral PTA described by Soda and colleagues had 1% detection threshold for X4 viruses [16]. Although the RL assay in DSP-Pheno could detect X4 laboratory strains present in concentrations as low as 0.3%, further studies are needed to apply the assay for the clinical use. DSP-Pheno may also be useful for the comparison of with GTA to improve the algorithm for the co-receptor usage of non-B subtypes.

## Conclusions

We described a new cell-fusion-based, high-throughput PTA for HIV-1, which would be suitable for in-house studies. Equipped with a two-way reporter system, RL and GFP, DSP-Pheno is sensitive and offers a short turnaround time. Although maintenance of cell lines and laboratory equipment for the assay is necessary, it provides a safe assay system without infectious viruses. With further validation against other conventional analysis, DSP-Pheno may prove to be a useful laboratory tool. The assay may be useful especially for the research on non-B subtype HIV-1 whose co-receptor usage has not been studied much.
